# Everyday helping is associated with enhanced mood but greater stress when it is more effortful

**DOI:** 10.1038/s41598-024-75261-z

**Published:** 2024-10-15

**Authors:** Ekaterina Pronizius, Paul A. G. Forbes, Anja C. Feneberg, Bianca Miculescu, Urs M. Nater, Giulio Piperno, Giorgia Silani, Ana Stijovic, Claus Lamm

**Affiliations:** 1 Department of Cognition, Emotion, and Methods in Psychology, Faculty of Psychology, University of Vienna, Liebiggasse 5, 1010 Vienna, Austria; 2 Department of Clinical and Health Psychology, Faculty of Psychology, University of Vienna, Vienna, Austria; 3 University of Vienna Research Platform “The Stress of Life (SOLE) – Processes and Mechanisms underlying Everyday Life Stress”, Vienna, Austria; 4 Faculty of Psychology and Educational Sciences, Psychological Sciences Research Institute, University of Louvain, Louvain-la-Neuve, Belgium; 5 Institute of Experimental Psychology, Heinrich Heine University, Düsseldorf, Germany

**Keywords:** Human behaviour, Health sciences

## Abstract

Our affective states can influence whether we help others and after helping we often experience improved affect. One important factor determining whether we help, is the amount of effort involved. Using an ecological momentary assessment approach across two measurement bursts (*N* = 803; *N* = 303), we investigated the affective antecedents and consequences of everyday helping in terms of participants’ self-reported momentary stress and mood valence, with a specific focus on the perceived amount of effort involved. Regardless of the amount of effort involved in helping, participants reported more positive mood valence after helping across both measurement bursts. In burst 2, this mood boosting effect of helping was strongest in those reporting lower mood prior to helping. In burst 1, we found a bidirectional relationship between stress and helping effort: the greater the effort involved in helping, the greater the perceived stress both before and after helping. Contrary to our preregistered hypotheses, changes in stress or mood valence did not precede helping regardless of the amount of effort involved. Our results support previous work linking helping to enhanced mood but suggest that when helping is more effortful it is both preceded and followed by greater stress. These findings have important implications for fostering and sustaining prosocial behaviours, especially when effort is involved.

## Introduction

Our current affective state can influence our behaviour towards others and it is often assumed that when “we feel good, we do good”^1–3^. For example, the induction of positive affect through a variety of methods, from receiving good news^4^ to thinking about the future^5^ to finding money^6^ have been shown to increase helping and prosociality [see Ref.^3^ for review]. After helping, people often experience a “warm-glow”^7^ which can motivate future prosocial acts^8^. Therefore, the relationship between helping and positive affect has been characterised as a positive feedback loop^9^.

This bidirectional relationship between momentary positive affect and prosocial behaviour has also been demonstrated in several ecological momentary assessment (EMA) studies^10–12^. EMA studies sample participants’ behaviour and experiences repeatedly, usually multiple times per day, as they occur in quasi real time in their natural environments^13^. Therefore, unlike laboratory studies that induce positive and negative affective states and determine their impact on subsequent behaviour, EMA studies can provide invaluable insights into the affective antecedents and consequences of helping in everyday life. However, not all EMA studies support the link between changes in mood and helping. For example, Taquet et al.^14^ used an EMA approach in over 28,000 participants and did not find an association. Here, we define helping as engaging in an act that benefits another person but comes at some cost to the helper—such as the investment of time, money, or other resources.

In addition to studies linking positive mood with helping, perceived stress can also modulate the likelihood of prosocial behaviour^15^. When exposed to an acute stressor participants show heightened neural responses to others’ pain, and these heightened responses predict subsequent prosocial decision-making in the dictator game^16^. Increased trust and sharing^17^ have also been demonstrated following experimental stress induction, although some studies have shown reduced prosociality following stress induction and others have shown no effects at all [Ref.^18^ for a recent menta-analysis]. Increased prosociality, or ‘tend-and-befriend’ behaviour, following stressor exposure could be a form of stress regulation. For example, providing support to another person reduces one’s own physiological responses to a stressor^19^. This stress buffering effect of helping was supported in a daily diary study by Raposa et al.^20^ who asked 77 participants to report their affective state, the number of stressors they had experienced, and the prosocial behaviours they had engaged in at the end of each day for two weeks. They found that the number of prosocial behaviours and stressors interacted to influence participants’ affective states, whereby engaging in a greater number of prosocial behaviours on a given day buffered the impact of stressors on negative affect.

One important factor which determines whether we help, and the potential affective consequences of helping, is the amount of effort involved. Lockwood et al.^21^ showed that participants display ‘prosocial apathy’ whereby they are less willing to help others if there are significant effort costs. Yet, most laboratory and field studies that have found links between affect and helping have not considered the role of effort. Positive affective states may not always lead to increased helping, especially if helping is costly in some way^22^. Participants in a positive affective state, compared to those in a neutral state, were less willing to help others if they thought that the act of helping would compromise their good mood^23,24^. Thus, if significant effort costs are involved in helping, individuals in a good mood could forgo helping to avoid anticipated reductions in their positive affect, as such reductions are often associated with effortful actions^25–27^. Similarly, people avoid feelings of empathy, if doing so entails effort costs^28^. Studies linking changes in stress to prosociality have tended to focus on tasks where participants incurred a financial cost but did not need to exert significant effort to be prosocial [Ref.^18^ for a recent menta-analysis]. Yet, prosociality under stress is dependent on the amount of effort involved^29^ and acute stress leads to a general avoidance of effortful actions^30,31^.

In sum, both positive affective states and perceived stress have been linked to increased helping (antecedents), and prosocial behaviour may reinforce existing positive moods or bring relief from aversive ones (consequences). However, the evidence from both laboratory and EMA studies have yielded mixed findings and the precise role of effort in modulating the link between mood/stress and helping remains to be understood. To this end, we used an EMA approach while COVID-19 lockdown restrictions were in place in April and May 2020 (burst 1; *N* = 803) and again in a further lockdown in November and December 2020 (burst 2; *N* = 303). Burst 2 included a subsample of the participants from burst 1 to test the temporal robustness of our effects in a separate lockdown. Five times per day for a seven-day period, participants reported their perceived stress and mood as well as whether they had helped since the last data entry. Crucially, participants rated the amount of effort involved in each helping episode.

Such an EMA approach allowed us to assess the momentary antecedents and consequences of helping as events unfolded in quasi-real time in participants’ natural environments^13^. This approach is also less vulnerable to recall bias associated with cross-sectional studies and enables the relationship between variables to be established in ecologically valid environments. During both measurement bursts, in March 2020 and November 2020, lockdown restrictions meant that participants were largely confined to their homes to limit the spread of the COVID-19. Participants were only allowed to leave their homes for a limited number of reasons^32^. In Austria, for example, non-essential businesses, such as retail and hospitality, were closed, and many organisations, including schools and universities, switched to remote operations. People were advised to stay at home unless they had to work jobs that they could not do from home, make necessary purchases (e.g., groceries), care for others, or seek medical treatment. Outdoor exercise (e.g. running, walking) was permitted but only alone or with members of the same household. Domestic and international travel was heavily restricted. Similar lockdown restrictions were in place in Italy and Germany during these periods.

Our hypotheses were preregistered (https://osf.io/gsvdf, *Hypotheses 5 A and 5 C*). First, we predicted that perceived stress/mood will be related to changes in helping. We aimed to test the “tend and befriend” hypothesis which predicts that perceived stress can result in prosocial consequences (e.g., increased helping) and that this increased prosociality reduces individuals’ own stress levels and improves their mood^15^. In contrast, the “fight or flight” hypothesis argues that perceived stress can lead to an increased urge to self-preserve, resulting in reduced prosocial behaviour^33^. In other words, we aimed to test whether changes in stress and mood were associated with the likelihood of subsequent helping. Whilst these hypotheses have predominantly been tested in laboratory studies which experimentally induced acute stress [see Ref.^18^ for a meta-analysis] or changes in mood [see Ref.^3^ for review], we aimed to test them using an EMA approach to see whether naturally occurring fluctuations in momentary stress and mood were related to an altered likelihood of subsequent helping.

Next, we predicted that the relationship between perceived stress/mood and helping would be modulated by perceived effort. Experimental work has shown that when participants are exposed to an acute stressor, they are less willing to exert effort^30^, especially when doing so for another person’s benefit^29^. Thus, we predicted that if changes in perceived stress/mood led to increased helping, this should only hold for helping associated with lower levels of perceived effort. In other words, perceived stress should show a negative association with the amount of perceived effort involved in subsequent helping and mood valence should show a positive association.

In terms of the consequences of helping involving different amounts of perceived effort, we did not preregister our hypotheses. Nevertheless, we also investigated whether the perceived effort involved in helping was associated with changes in stress/mood after helping. For example, we tested whether the greater effort involved in helping resulted in higher subjective stress levels and/or reductions in positive mood.

## Results

Descriptive statistics for momentary stress, mood valence, number of helping episodes and amount of effort involved across two study bursts are reported in Table 1.

**Table 1:** Descriptive statistics for momentary stress, mood valence, number of helping episodes and amount of effort involved across two study bursts.

	Burst 1		Burst 2	
	*M* (*SD*)	*N*	*M* (*SD*)	*N*
Momentary stress	30.26 (17.93)	803	28.03 (18.15)	303
Mood valence	63.27 (14.70)	803	63.37 (14.88)	303
Helping data entries	3.37 (4.86)	803	3.41 (4.89)	303
Helping effort	35.89 (18.81)	527*	34.95 (18.52)	200*

Scales’ range: momentary stress (0–100), mood valence (0–100), helping data entries (0–35), and helping effort (0–100).

*M* mean*, SD* standard deviation.

*Only helpers.

To gain an insight into the types of helping people engaged in, participants in burst 2 provided an example of their most recent helping episode in an additional online questionnaire at the end of the study (see *Methods*). This revealed that helping was largely directed towards close others: a friend (29.03%), a parent (17.20%), a relative (15.59%), a partner (15.05%), a child (12.37%), a colleague (8.60%), or charity (2.15%). We also classified helping into the following categories: helping within the household or chores (32.78%); helping with work, study, or teaching a skill (23.65%); comforting (14.12%); caregiving (9.96%); giving advice (7.88%); sharing resources (6.22%); and accompanying someone (5.39%). The classification was data- and theory-driven^34–38^ and performed by two independent raters [EP and BM; final agreement 96%].

## More positive mood valence after helping

### Antecedents of helping (preregistered)

In terms of the potential impact of stress on helping, perceived stress in the previous data entry was not associated with a greater likelihood of helping in the subsequent data entry in burst 1 (*β* = 0.005, SE = 0.003, *p* = 0.089) or burst 2 (*β* = 0.000, SE = 0.005, *p* = 0.984). Similarly, mood valence was not associated with differences in the likelihood of helping in either burst 1 (*β* = 0.000, SE = 0.004, *p* = 0.944) or burst 2 (*β* = 0.012, SE = 0.008, *p* = 0.124).

### Consequences of helping (preregistered)

The analysis revealed no significant effect of helping on subsequent momentary stress in either burst 1 (*β* = 0.348, SE = 0.576, *p* = 0.546) or burst 2 (*β* = -1.035, SE = 1.117, *p* = 0.355). Yet, having helped since the previous data entry was associated with more positive mood valence (burst 1: *β* = 1.824, SE = 0.472, *p* < 0.001, *d* = 0.432; burst 2: *β* = 1.963, SE = 0.758, *p* = 0.010, *d* = 0.085). As we controlled for the level of mood valence reported in the data entry before helping took place, the effect of helping on subsequent mood was not simply the result of participants having greater mood valence in the data entry before helping.

### Interaction effects

We found no significant interactions between perceived stress from the previous data entry and helping on subsequent momentary stress in either of the study bursts (burst 1: *β* = -0.055, SE = 0.028, *p* = 0.055; burst 2: *β* = -0.067, SE = 0.048, *p* = 0.164).

**Fig. 1:**
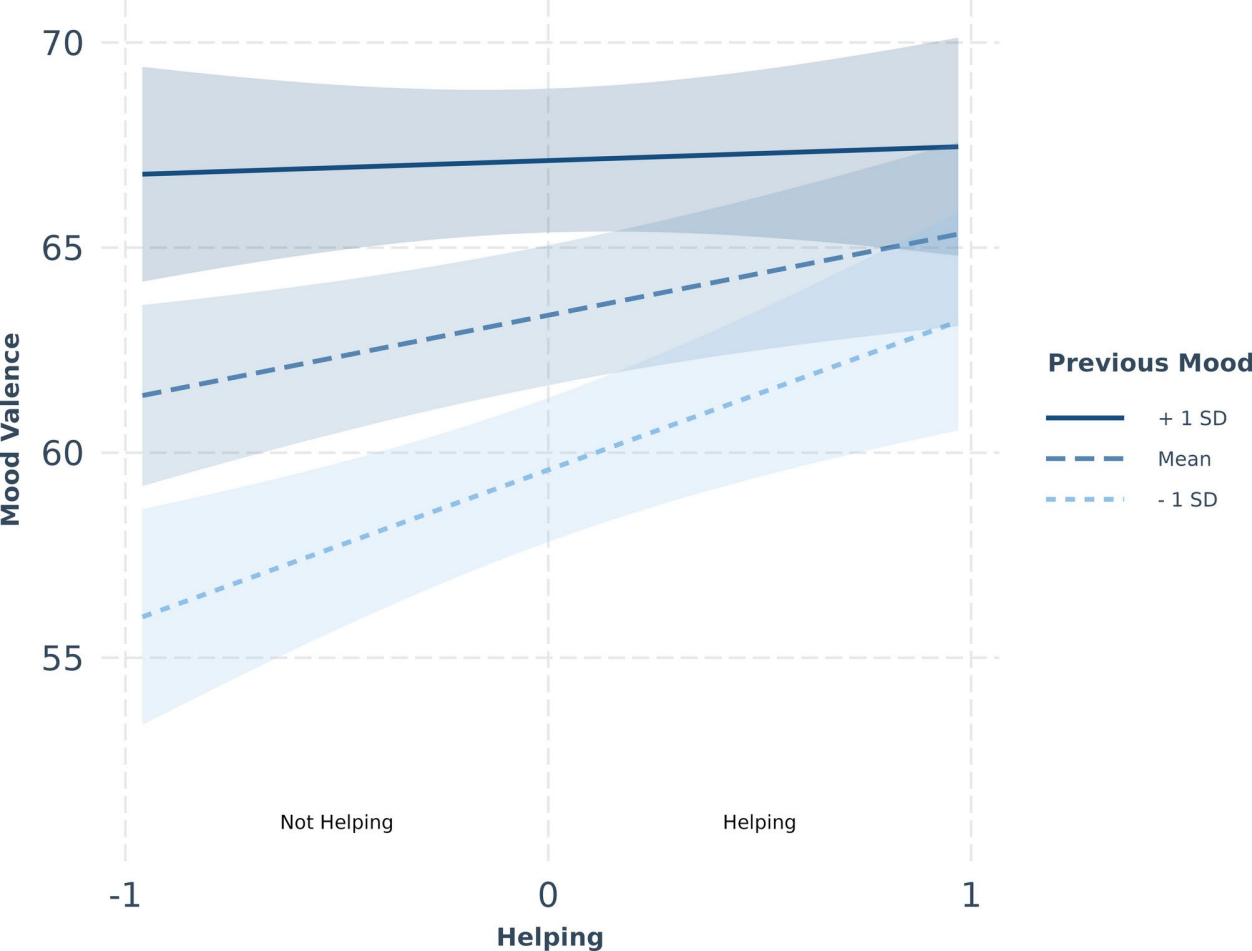
Helping and momentary mood valence in burst 2. The slopes showing the relationship between helping and momentary mood valence in burst 2 at different levels of mood valence in the previous data entry. The ribbons represent 95% confidence intervals. As the number of helping episodes varied across participants, this variable was participant mean-centered and thus represented as a continuous variable (*not helping* < 0; *helping* > 0). The *interact_plot* function from the R package *jtools*^39^ was used to create the plot.

We found a significant interaction between mood valence from the previous data entry and helping on subsequent momentary mood valence in burst 2 (*β* = -0.117, SE = 0.050, *p* = 0.019) but not burst 1 (*β* = -0.046, SE = 0.030, *p* = 0.128). The model for burst 2 did not converge with a random slope for helping so the results should also be interpreted with caution. Nevertheless, simple slope analysis showed that lower levels (−1 *SD, β* = 3.734, SE = 1.044, *p* < 0.001) and mean levels of previous mood valence (*β* = 2.040, SE = 0.755, *p* = 0.007) led to increased momentary mood valence after helping compared to higher levels of previous mood valence, which were not significant (*β* = 0.347, SE = 1.043, *p* = 0.739). Thus, if participants showed reduced or average mood valence in the previous data entry, then helping improved mood. Whereas, if participants were already in an above average mood, helping did not change subsequent mood (Fig. 1).

The significant results are summarized in Table 2; all results can be found in Supplementary 4.

**Table 2:** Helping.

Model	Study burst	Description	Estimate (β)	Standard error (SE)	p-value
1	burst 1	Helping on momentary mood valence	1.824	0.472	< 0.001
2	burst 2	Helping on momentary mood valence	1.963	0.758	0.010
3	burst 2	Interaction effect of helping and previous mood valence on momentary mood valence	-0.117	0.050	0.019

Models 1 and 2: momentary mood valence ~ helping + previous mood valence + free time + time of day + (helping + free time | subject).

Model 3: momentary mood valence ~ helping * previous mood valence + free time + time of day + (time of day + free time | subject).

## Greater perceived stress before and after more effortful helping

### Antecedents of helping effort (preregistered)

In burst 1, higher perceived stress from the previous data entry was positively associated with the amount of effort involved in subsequent helping (*β* = 0.067, SE = 0.028, *p* = 0.021, *d* = 0.388; burst 2: *β* = 0.038, SE = 0.041, *p* = 0.352). Across both study bursts, perceived mood valence from the previous data entry was not significantly associated with the amount of effort involved in subsequent helping (burst 1: *β* = -0.029, SE = 0.037, *p* = 0.434; burst 2, *β* = -0.064, SE = 0.054, *p* = 0.237).

### Consequences of helping effort (not preregistered)

We found that the greater the effort involved in helping was, the greater the perceived momentary stress after helping in burst 1 (*β* = 0.088, SE = 0.031, *p* = 0.005, *d* = 0.547), but not burst 2 (*β* = 0.026, SE = 0.049, *p* = 0.586). The analysis revealed no significant effect of helping effort on momentary mood valence (burst 1: *β* = -0.041, SE = 0.026, *p* = 0.124; burst 2: *β* = -0.045, SE = 0.038, *p* = 0.235).

The significant results are summarised in Table 3; all results can be found in the Supplementary 4.

**Table 3:** Helping effort.

Model	Study burst	Description	Estimate (β)	Standard error (SE)	p-value
1	burst 1	Stress from previous data entry on helping effort	0.067	0.028	0.021
2	burst 1	Helping effort on momentary stress	0.088	0.031	0.005

Model 1: helping effort ~ previous stress + free time + time of day + (previous stress + free time | subject). Model 2: stress ~ helping effort + previous stress + free time + time of day + (helping effort + free time | subject).

### Effects of COVID-19-related concerns, day of the week, and social interactions within helping episodes

To account for the potential confounding effects of COVID-19-related concerns, day of the week, and social interactions, we conducted additional analyses. First, we included COVID-19-related concerns (person mean-centered), free time, and time of day as predictors, with helping or helping effort as the dependent variable. COVID-19-related concerns was not a significant predictor of either helping (burst 1: *p* = 0.070, burst 2: *p* = 0.569) or helping effort (burst 1: *p* = 0.487, burst 2: *p* = 0.850).

Next, we investigated the effects of the day of the week on helping, while also controlling for time of day and free time. Across both study bursts, individuals were less prone to help during weekends (burst 1: β = −0.159, SE = 0.057, *p* = 0.005; burst 2: β = −0.318, SE = 0.096, *p* < 0.001).

Finally, we looked at the role of social interactions and helping as having a social interaction rather than helping could have accounted for our effects. It is worth noting that less than 10% of helping episodes in burst 1 (*n* = 256 out of 2704 helping episodes) and less than 7% of helping episodes in burst 2 (*n* = 71 out of 1033 helping episodes) did not involve social interaction. Further, only 131 participants in burst 1 (from 528 participants who helped at least once) and 49 participants from burst 2 (from 201 helpers) helped without social interaction. Therefore, this analysis may have had limited power to detect effects. Nevertheless, when we directly compared helping with a social interaction to helping without a social interaction, we did not find any significant differences in their effects on mood valence after helping (burst 1: β = 3.427, SE = 2.015, *p* = 0.092; burst 2: β = −4.702, SE = 3.064, *p* = 0.126).

Thus, whilst we did not find any significant differences between helping with and without a social interaction on subsequent mood valence a lack of power could drive this null finding. Thus, distinguishing between the effects of social interaction versus the specific effects of helping should be a key aim for future studies.

## Discussion

Using an EMA approach, we investigated the affective antecedents and consequences of everyday helping with a particular focus on the amount of perceived effort involved. Across both measurement bursts, we found that participants reported more positive mood valence after helping compared to when they had not helped someone. In burst 2, we found that this effect was strongest when participants reported lower mood valence in the previous data entry. When helping involved more perceived effort, participants reported greater stress before and after helping, although this effect was only seen in burst 1. In other words, helping was followed by enhanced mood, but also by greater stress when it was perceived as more effortful.

We did not find a relationship between participants’ perceived stress or mood and the probability of subsequent helping. This suggests that helping was independent of stress levels and mood prior to helping. These results are in contrast to the view that when “we feel good, we do good”^1–3^. Moreover, they do not support the predictions of the “tend and befriend” hypothesis^15^, which predicts an increase in prosocial tendencies under stress.

We predicted that higher levels of perceived stress would be associated with lower effort helping, but we found the opposite: greater perceived stress was associated with higher perceived effort involved in helping, and participants also reported more stress after more effortful helping. Our prediction was based on laboratory findings demonstrating that effortful prosocial behaviour under acute stress is dependent on the amount of effort involved^29^ and acute stress leads to a reduced willingness to exert effort^30,31^. One potential explanation to account for this discrepancy is that the vast majority of helping in our study was likely directed towards close others (e.g., family, and friends), whereas our laboratory study investigated helping towards unknown others^29^. Thus, effort costs may not have a differing impact under stress when helping is directed towards close others. Moreover, the intended goal of the effort required may also be important. For example, a recent study showed that when effort was directed towards safety goals, acute stress led to greater effort^40^.

Our findings concerning the momentary consequences of helping support previous studies demonstrating the benefits of helping in terms of increasing positive affect^7,8,41^ and improving well-being^11,42^. We extend these findings in two ways. First, we showed in burst 2 that the potential mood enhancing effect of helping was strongest in those with lower levels of mood valence prior to helping. This has important implications for interventions aimed at improving affective states through acts of helping^43,44^. For example, prosocial acts have been shown to reduce intrusive traumatic memories^45^ and improve positive emotions during the COVID-19 pandemic^46^. Moreover, most laboratory studies have focused on the immediate effects of helping [e.g., Ref.^7^], here we show that helping was associated with affective benefits lasting over longer periods suggesting that a “warm glow” could persist for several hours after helping in daily life.

Secondly, while helping generally improves mood, the impact of helping on affect can vary based on the perceived effort involved. Specifically, in burst 1, more effortful helping was associated with increased stress following helping. If higher helping effort is associated with increased stress levels, this could at least partially explain why people avoid effortful helping^21^. Shaw et al.^47^ found that participants actively avoided feelings of empathy for a man experiencing homelessness, if they knew that helping him in the future would involve high financial costs compared to participants who were led to believe that helping involved low costs. People may anticipate the negative consequences of costly helping for their perceived stress levels and thus avoid it. This finding has important implications for encouraging people to help others. For example, in terms of volunteering, if the perceived effort costs are low, such as one-off volunteering opportunities rather than a longer-term commitment, this is likely to encourage prosocial behaviour^48^.

How do we reconcile our findings of increased perceived stress following effortful helping with examples of effortful helping in everyday life? If effortful helping results in greater stress, why do people run marathons for charity or give up their free time to volunteer? Here, it may be important to distinguish between the relatively short-term, momentary effects of effortful helping, which is what we assessed in the current study, and the longer-term effects of effortful helping. Inzlicht et al.^49^ have argued that one’s own effort is often appreciated more and valued more highly retrospectively (e.g. the IKEA effect;^50^). Thus, effortful helping may be stressful in the moment but could bring longer term affective benefits upon reflection. Moreover, we need to consider that many acts of helping (e.g. running a marathon for charity, volunteering) are driven by a diverse range of motives^51^ rather than simply changing or avoiding certain momentary affective states.

### Limitations

Data was collected during COVID-19 lockdowns; therefore, opportunities to help others may have been limited as people were largely confined to their homes. This is particularly important when considering that participants predominantly helped close others. For example, planned helping, such as volunteering, which is typically directed towards less close others, was limited due to the lockdown restrictions. Aydinli et al.^52^ suggest that the determinants of these different types of helping (i.e., spontaneous helping of close others vs. planned helping of strangers) are distinct. Therefore, it is likely that their affective precedents and consequences also show important differences.

Further, participants’ daily routines and weekly structure were affected by the lockdown with many forced to work from home. Previous studies found that daily helping behaviours (and other interactions with strangers) were more frequent on weekends and least frequent mid-week^53^. However, our analysis revealed the opposite pattern, with helping being less frequent during the weekend, which could be explained by the effects of the lockdown. Future studies could model cyclic patterns and other weekly trends to investigate the influence of weekdays versus workdays on helping behaviour toward strangers and family members.

Additionally, the participants in our study reported being more stressed and depressed during this period compared to age-matched norms (Supplementary 1). Thus, it could be that the mood-boosting effects of helping were particularly pronounced during this period and thus participants sought out opportunities to help others to combat their low mood. Indeed, studies have reported increased prosocial behaviour (“catastrophe compassion”) under the threat of COVID-19^54^. Similarly, thinking about one’s mortality has been shown to boost prosocial behaviour (“Scrooge effect”^55^) and there is some evidence to suggest that thoughts about mortality increased during the COVID-19 pandemic^56^. Together, multiple factors specific to the time period during which our data were collected could have impacted the results. Thus, the results will need to be replicated outside of this specific context.

Further, to differentiate the effects of helping from those of having a social interaction, we conducted additional analyses focusing on the impact of helping on mood valence during the two bursts. Our findings indicate that there was no significant difference in mood improvement between helping with a social interaction and helping without one. Yet, the analysis may have had limited power to detect any effects due to the small number of helping episodes without social interaction (less than 10% of all helping episodes did not involve a social interaction), and follow-up studies will be needed to disentangle the effects of helping from those of having a social interaction. For example, this could be done by comparing online charitable donations to those done in person whilst also controlling for important factors such as anonymity^57^.

We observed an effect of helping effort on momentary stress in burst 1, but not in burst 2. While both bursts had substantial sample sizes (803 participants in burst 1 and 303 in burst 2), the difference in findings may be due to the relatively lower number of helping episodes in burst 2. Additionally, the second lockdown, during which burst 2 was conducted, differed from the first in several key aspects, such as changes in public compliance, adaptation to restrictions, and the overall psychological fatigue associated with the pandemic. These factors may have influenced the generalizability of the findings, potentially weakening the observed effects in the second burst. Again, future studies will need to determine the robustness of this effect.

Finally, participants self-reported the level of effort involved in helping. Stressed participants may have overestimated the effort costs involved in helping, as people evaluate effort costs differently under stress^29,30^, which could have masked potential effects. Thus, future EMA studies are needed to support the current findings. Such studies could assess effort more objectively (i.e., via financial or physical costs measured with wearable technologies, e.g., accelerometers^58,59^) and, ideally, before and after engaging in helping, utilizing event-based measurements.

## Conclusion

We did not find that prior changes in stress or mood were associated with a greater likelihood of helping, thereby challenging previous findings from laboratory studies. However, in terms of the consequences of helping, we found that helping was associated with enhanced mood but also with greater stress when it was perceived as more effortful. These findings have important implications for encouraging prosocial behaviour and raise the possibility that there are different short-term and long-term affective responses to effortful helping. By increasing our understanding of the antecedents and consequences of everyday helping, particularly helping involving more effort, our results could prove useful in fostering and sustaining prosocial behaviour with potential benefits for both the helper themselves and the person being helped.

## Methods

### Participants

The present study was part of a larger project carried out within the context of the COVID-19 pandemic^60–63^, which consisted of two study bursts. The full list of the variables associated with this project can be found here https://osf.io/4tpvd. The sample was recruited via a range of different channels including an existing database (Vienna Cognitive Science Hub, consisting of individuals from a general population, organised and recruited with the software hroot, Ref.^64^), advertisements in online media outlets, by word of mouth, and social media. To take part in the first study burst, participants had to have an Android device to use the app, be 18 years old or older, and be residing in Austria, Germany, or Italy, as similar lockdown restrictions were in place across these countries during the data collection. All participants in both bursts had to provide data in at least 50% of the data entries (preregistered completion rate cut-off). For the second study burst, we invited those participants who provided data in at least 50% of data entries in the first study burst and were residing in Austria. We were not able to invite participants from Italy or Germany due to logistical reasons.

The final sample of burst 1 comprised 803 participants (522 were in Austria; 251 in Italy; 30 in Germany; *M*_*age*_ = 31.87, *SD*_*age*_ = 11.86; 559 women), and burst 2 included 303 participants (all in Austria; *M*_*age*_ = 34.05, *SD*_*age*_ = 13.15; 238 women). The mean completion rate (the proportion of prompts to which data was provided) was 78% in burst 1 and 76% in burst 2. Therefore, in both bursts, participants provided data in response to approximately 27 prompts out of a maximum of 35 (5 prompts per day over 7 days). Further information on participants’ attrition, data cleaning, and details on completion rates and response latency as well as a comparison of the sample to normative questionnaire values can be found in the supplementary materials (Supplementary 1–3).

#### Study burst 1

Out of 803 eligible participants, 276 were “non-helpers”. Comparisons between the helpers vs. the non-helpers (see Supplementary 1, *Table S1*) showed that the helpers reported more social exchanges (*p* < 0.001), more caregiving responsibilities (*p* < 0.001), greater average mood valence (*p* = 0.028), higher values on a prosociality scale (*p* = 0.013), were less lonely (*p* = 0.014) and had less COVID-19 related concerns (*p* = 0.009). The proportion of males was significantly higher in the non-helpers (*p* = 0.023) and this group was also younger (*p* = 0.025).

#### Study burst 2

Out of 303 eligible participants, 103 were “non-helpers.” Helpers compared to non-helpers from the second lockdown (see *Table S1*) reported more social exchanges (*p* = 0.008), had higher values on a prosociality scale (*p* = 0.036) but had less free time than non-helpers (*p* = 0.009).

We also run a comparison between both groups of helpers (burst 1 and burst 2). Helpers from burst 1 had less caregiving responsibilities (*p* < 0.001), greater average energetic arousal (*p* < 0.001) and higher proportion of free time (*p* < 0.001) than helpers from burst 2. The proportion of males was significantly higher in the helpers from burst 1 compared to burst 2 (*p* = 0.050).

### Measures

Momentary perceived stress was assessed with a single item “*At the moment, I feel stressed*” with a visual analogue scale (VAS) ranging from 0 (*not at all*) to 100 (*very much*). Mood valence was measured with an adapted multidimensional mood questionnaire^65^, which had been validated for use in EMA studies^66^. The mood valence scale comprised two bipolar items (*unwell-well* and *dissatisfied-satisfied* items) and the aggregated score ranged from 0 to 100 with higher values corresponding to higher levels of mood valence. In our preregistration, we also stated that we would investigate calmness and energetic arousal as additional mood states. For brevity, we do not report these findings in the main manuscript, but these are reported and discussed in Supplementary 5.

Helping was measured with a single dichotomous item “*Since the last data entry, have you helped or supported someone?*” (0 = *no*, 1 = *yes*). If the participants indicated having helped since the last entry, they were additionally asked a) whether the person who received help/support lives in the same household or not (0 = *no*, 1 = *yes*); and b) how effortful this helping or support was (variable *helping effort* ranging from 0 = *not at all* to 100 = *very much)*.

#### Types of helping behaviour

At the end of burst 2, participants provided details about their most recent helping episode by answering the following questions: “*Think about the last time/situation in which you helped or supported another person/persons. 1) When was the last time you helped/supported someone*? 2) *How did you help/supported? Please describe briefly in 4–5 keywords;* 3) *Who was the person(s) you helped/supported*?;” and finally, “*How effortful was this helping or support?”* (0 = *not at all* to 100 = *very much*).

### Data collection

*Burst 1.* Data was collected between 1st April and 24th April 2020 (German-speaking sample) and between 13th April and 5th May 2020 (Italian-speaking sample) during the first wave of the COVID-19 pandemic as participants were under comparable national lockdown restrictions in Austria, Italy, and Germany. *Burst 2.* Data was collected between 23rd November and 6th December 2020 in Austria during the second national lockdown.

**Fig. 2:**
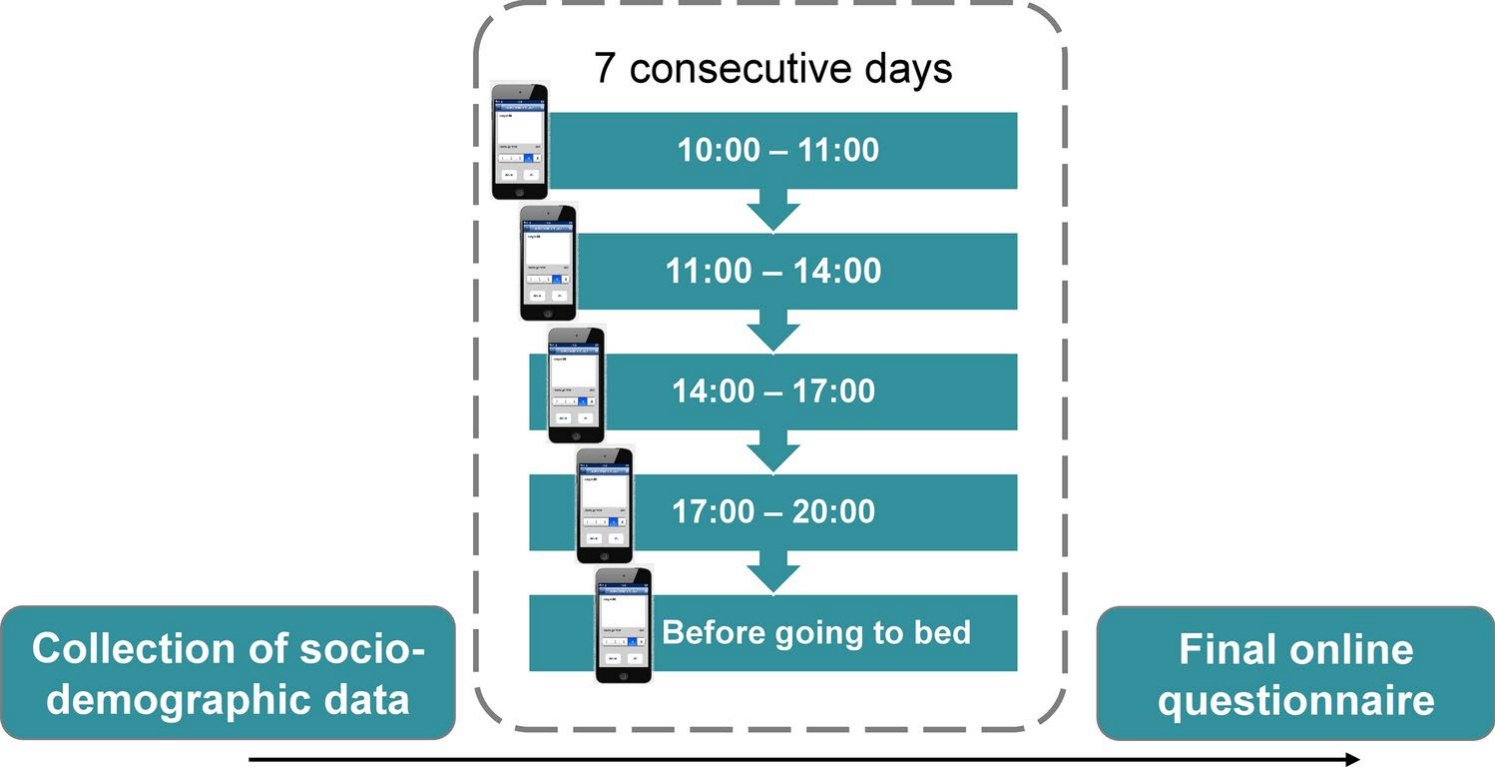
The study protocol and measures. EMA items of interest: *“At the moment, I feel stressed”* (VAS: 0 = *not at all *– 100 = *very much*); Mood valence measure: mean of two items: *“At the moment, I feel unwell-well”* and *“*…*dissatisfied-satisfied”* (VAS: 0 = *not at all *– 100 = *very much*); Helping measure: “*Since the last data entry, have you helped or supported someone?”* (0 = *no*, 1 = *yes*); “*If yes: how effortful this helping or support was?”* (VAS: 0 = *not at all* – 100 = *very much*).

Participants who met the inclusion criteria received a customised link for the app “movisensXS” (movisens GmbH, Karlsruhe, Germany). The data collection lasted nine days for each participant (Fig. 2). On the first day, participants provided digital informed consent, their socio-demographic data (e.g., age, gender, place of residence) and answered several questions related to the COVID-19 pandemic and its impact.

Beginning on the following day, for seven consecutive days, we repeatedly collected multiple data entries of participants’ momentary stress and mood as well as their recent behaviours. We used an EMA approach where participants were prompted via a pre-programmed smartphone app to submit data five times per day for seven successive days. The prompts appeared semi-randomly over the course of the day (between 10 a.m.–11 a.m., 11 a.m.–2 p.m., 2 p.m.–5 p.m., 5 p.m.–8 p.m.). The final fifth data entry was self-initiated before going to sleep.

For each data entry (see Fig. 2), participants reported how stressed they currently were, their momentary mood valence (two bipolar items: *unwell-well* and *dissatisfied-satisfied* items)^65,66^ and indicated whether they had helped or supported someone since the last data entry and how effortful this was. In all analyses, we controlled for the activity participants were currently engaged in and the time of day as both influence stress and mood^60^ as well as the probability of helping (see *Methods*).

On the ninth day, participants completed the final online questionnaire (SoSci Survey GmbH, Munich, Germany) and received monetary compensation (€20 burst 1, €30 burst 2). The study was approved by the Ethics Board of the University of Vienna (ref. num. 00553) and was conducted in accordance with the latest revision of the Declaration of Helsinki. Informed consent was obtained from all participants.

### Statistical analysis

We used multilevel models with observations on level 1 nested within individual participants on level 2. The n values for each statistical analysis: (1) the models with the key variable *helping* converged with *N* = 803 participants and 14,183 observations in burst 1, and *N* = 303 participants and 5,117 observations in burst 2; and (2) the models with the key variable *helping effort* converged with *N* = 451 participants and 1591 observations in burst 1, and *N* = 163 participants and 592 observations in burst 2. The analysis was conducted using the *lme4* and *glmmTMB* packages in R^67–69^. The models included random slopes and random intercepts and were kept maximal whenever possible^70^. The random effects structure was built as follows: first, we included the random slopes of key variables of interest (stress/mood valence or helping). Next, we increased the complexity of the random effects structure by adding variables in sequence. We began with free time, given its significant impact on momentary stress and mood^60^, and subsequently included time of day.

The linear mixed models were fitted by applying the restricted maximum likelihood estimation and Satterthwaite’s methods^67^. The generalized linear mixed models were fitted using maximum likelihood estimation via “TMB” (Template Model Builder), binomial family. Level 1 predictors (except for time of day, which was centered at 10 a.m.) were participant mean-centered^71^. We included the variables *free time* (0 = *no*, 1 = *yes*) and *time of day* as predictors in all models to control for time of day and the activity participants were engaged in during the time of the assessment. Interactions were explored with simple slope analyses using the *sim_slopes* function from the R package *interactions*^72^.

### Antecedents and consequences of helping and helping effort

#### Antecedents

In these models, *helping* or *helping effort* was a dependent variable and the respective stress or mood valence from the previous data entry was a continuous predictor (see Supplementary 4).

#### Consequences

To investigate the consequences of helping, we conducted multilevel modelling in a stepwise manner. First, we tested separate models with helping as a predictor and stress or mood valence as a dependent variable. To ensure that the effect of helping on stress or mood valence was not simply the result of participants having greater stress or mood valence in the data entry before helping, we controlled for it by including the respective measure from the previous data entry. In the second step, we built an *interaction term* between previous stress or mood valence and helping. With this interaction term, we tested our prediction that the effect of helping on momentary stress and mood valence varies depending on previous levels of stress and mood valence.

To investigate the consequences of helping effort as a predictor, we run separate models with momentary stress or mood valence as a dependent variable. The stress or mood valence from the previous data entry was added as a control variable.

Additionally, we included *free time* (0 = *current data entry while working/studying* or 1 = *during free time*), and *time of day* (time passed since 10 a.m.) as covariates in all models. This allowed us to determine the antecedents and consequences of helping / helping effort independent of time of day or the activity participants were engaged in. Accounting for time of day and participant activities is common practice in ecological momentary assessment research, especially when investigating mood states^13,73,74^**.**

### Effects of COVID-19-related concerns, day of the week, and social interactions within helping episodes

To address the potential confounding effects of other variables, we conducted additional analyses with COVID-19-related concerns, day of the week, and social interactions during helping episodes. First, we included COVID-19-related concerns (person mean-centered), free time, and time of day as predictors, with helping or helping effort as the dependent variable. COVID-related concerns were operationalized as the mean of four items: concerns 1) for personal health, 2) for the health of significant others, 3) for personal finances, and 4) for personal relationships. Participants rated their level of agreement with each statement on a scale from 0 (*strongly disagree*) to 100 (*strongly agree*) once, on the first day of the study.

Next, we investigated the effects of the day of the week on helping (0 = *work day*, 1 = *weekend*), while also controlling for time of day and free time. Finally, we examined the distinct effects of social interaction compared to the specific effects of helping on subsequent mood valence (the outcome with significant results).

## Supplementary Information

Supplementary Information 1.

## Data Availability

The project’s preregistration can be found here https://osf.io/gsvdf. Data and code associated with the present study have been made publicly available at the OSF repository and can be accessed at https://osf.io/6n5zj/.
