# Supplementary material for: Everyday helping is associated with enhanced mood but greater stress when it is more effortful
Source: Sci Rep. 2024 Oct 15;14:24120. doi: 10.1038/s41598-024-75261-z (PMC11480085; doi:10.1038/s41598-024-75261-z)
Supplement: Supplementary file 1 — Supplementary Information 1. [file 41598_2024_75261_MOESM1_ESM.pdf]

SUPPLEMENTARY MATERIALS

Supplementary 1. Sample comparison to normative values

Table S1. Samples description

	Burst 1		Burst 2	
	Helpers	Non-helpers	Helpers	Non-helpers
	Value *M* (*SD*)	Value *M* (*SD*)	Value *M* (*SD*)	Value *M* (*SD*)
Prosocialness[a]	58.43 (9.59)	56.59 (9.37)	57.03 (9.87)	54.45 (10.17)
Caregiving responsibilities	.22 (.41)	0.11 (0.32)	0.35 (0.48)	0.31 (0.47)
Employment status	.91 (.28)	0.89 (0.31)	0.93 (0.26)	0.94 (0.24)
Mental health condition	.14 (.35)	0.11 (0.32)	0.17 (0.38)	0.16 (0.36)
PSS[b]	18.83 (7.19)	18.91 (7.63)	18.92 (7.25)	18.4 (7.25)
Loneliness[c]	37.62 (9.50)	39.47 (10.01)	38.33 (10.21)	36.52 (9.30)
Depression[d]	8.05 (5.14)	8.43 (5.27)	8.55 (5.51)	8.32 (5.17)
Momentary stress	29.77 (17.69)	31.2 (18.37)	28.79 (17.76)	26.54 (18.86)
Mood valence	64.09 (14.61)	61.7 (14.77)	62.64 (15.32)	64.78 (13.94)
Calmness	62.11 (15.35)	60.6 (15.8)	60.63 (16.31)	61.67 (15.43)
Energetic arousal	51.01 (10.60)	50.48 (11)	48.00 (11.36)	47.47 (11.01)
Helping data entries	5.13 (5.19)	-	5.17 (5.21)	-
Helping effort	35.89 (18.81)	-	34.95 (18.52)	-
Proportion of freetime	.53 (.22)	0.51 (0.24)	0.46 (0.22)	0.53 (0.21)
Social exchange	.75 (.21)	.68 (.25)	.73 (.21)	.66 (.25)
COVID-19-related concerns[e]	48.68 (17.85)	52.39 (19.59)	50.86 (18.85)	50.42 (15.79)

Note. The following variables were assessed only once, on the first day of the study: caregiving responsibilities (0 = no, 1 = yes, e.g., children, elderly, others), employment status (0 = unemployed, 1 = employed), mental health condition (0 = absent, 1 = present); e = The mean of four items measuring COVID-19-related concerns [scale range 0 = strongly disagree - 100 = strongly agree]: I am concerned about the impact of the virus 1) on my health; 2) on the health of the most important people in my life; 3) on my personal financial status; 4) on my relationships with the most important people in my life.

The following variables were assessed only once on the final day of the study by using questionnaires: a = Prosocial Scale [1, scale range 16-80], b = Perceived Stress Scale [PSS-10 2, scale range 0-40]; c = the UCLA Loneliness Scale [3, scale range 20-80]; d = Patient Health Questionnaire [4,5, scale range 0-27].

Ecological momentary assessment scales: momentary stress (0-100), mood valence (0-100), energetic arousal (0-100), total helping data entries (0-35), helping effort (0-100), proportion of free time (0-1), proportion of social exchange (0-1).

The present sample reported higher levels of chronic stress (burst 1: mean PSS = 18.86, *SD* = 7.34, burst 2: mean PSS = 18.75, SD = 7.24) compared to the mean PSS of 12.74, *SD* = 6.67, in the group of 20- to 39-year-olds from the representative German community sample [2]. Loneliness levels of the present sample did not differ from the average loneliness in a normative sample (burst 1: mean loneliness score 38.25, *SD* = 9.71; burst 2: mean loneliness score 37.73, *SD* = 9.94 as compared to the mean of 40.8 in students in [3]). 21% of the participants from burst 1 and 21% of the participants from burst 2 reported moderate (PHQ-9 cut-off 10-14) depressive symptoms. Further, 11% of the participants from burst 1 and 14% of the participants from burst 2 reported severe depressive symptoms (cut-off 15-27, as in [4]). This number is notably higher than 5.6% of participants with moderate and severe symptoms in a German normative sample collected before the pandemic [6].

Supplementary 2. Participant attrition and data cleaning

Burst 1. First, we discarded invalid observations (*n* = 134). The observations were classified as invalid if (a) the time point of the self-initiated data entry before going to sleep was after 6 a.m., (b) the response latency (time elapsed since the alarm and starting to respond) exceeded 1 hour, and (c) the response duration (time elapsed from starting until finishing the response) exceeded 20 minutes.

Next, we excluded the participants (*n* = 148), who a) had less than a 50% completion rate, b) were under the age of 18 years, or c) resided outside of Austria, Germany, or Italy.

Burst 2. First, *n* = 65 invalid (as defined above) observations were discarded. Next, we excluded the participants (*n* = 54), who had less than a 50% completion rate or were under the age of 18 years.

Supplementary 3. Details on compliance rates and response latency

Burst 1. Overall, the completion rate in the final sample (*n* = 803) was good, with a mean of 78% (*SD* = 13%) of accepted data entries. On the participant level, completion rates ranged from 51.4% to 100%. The mean response duration was 2:29 minutes (*SD* = 1.79). Descriptively, taking the variable stress as an example, most missing entries occurred at the first data entry of the day (first prompt between 10-11 am; *n* = 1,379) and the least missing entries occurred at the self-initiated bedtime data entry (*n* = 1,072). Furthermore, the completion rate deteriorated throughout the seven days of the study (*n* = 674 missing entries on the first study day; *n* = 1,300 missing entries on the last day).

Burst 2. The completion rate in the helpers from burst 2 (*n* = 303) had a mean of 76% (*SD* = 13%) of accepted data entries. On the participant level, completion rate ranged from 51% to 100%. The mean response duration was 2:82 minutes (*SD* = 2.08). Similarly, to burst 1, most missing entries occurred at the first data entry of the day (*n* = 542) and the least missing entries occurred at the self-initiated bedtime data entry (*n* = 418). Furthermore, the completion rate deteriorated throughout the seven days of the study (*n* = 275 missing entries on the first study day; *n* = 519 missing entries on the last day).

Supplementary 4. Statistical models and their outcomes.

The key variable of interest is shown in **bold** and the statistical results for this variable are shown in the *model output* column.

Table S2. Detailed summary of results reported in the main manuscript.

Helping

Antecedents of helping

Burst	*Statistical model (R code)*	*Model output for the key predictor*
1	helping ~ **previous stress** + freetime + ematime + (**previous stress** + freetime | subject)	*β* = 0.005, SE = 0.003, *p* = .089
2	helping **~ previous stress** + freetime + ematime + (**previous stress** + ematime | subject)	*β* = 0.000, SE = 0.005, *p* = .984
1	helping ~ **previous mood valence** + freetime + ematime + (**previous mood valence** + freetime + ematime | subject)	*β* = 0.000, SE = 0.004, *p* =.944
2	helping **~ previous mood valence** + freetime + ematime + (**previous mood valence** + freetime | subject)	*β* = 0.012, SE = 0.008, *p* =.124

Consequences of helping

1	momentary stress ~ **helping** + previous stress + freetime + ematime + (**helping** + freetime + ematime | subject)	*β* = 0.348, SE = 0.576, *p* = .546
2	momentary stress ~ **helping** + previous stress + freetime + ematime + (**helping** + freetime + ematime | subject)	*β* = -1.035, SE = 1.117, *p* = .355
1	momentary mood valence ~ **helping** + previous mood valence + freetime + ematime + (**helping** + freetime | subject)	***β* = 1.824, SE = 0.472, *p* < .001**
2	momentary mood valence ~ **helping** + previous mood valence + freetime + ematime + (**helping** + freetime | subject)	***β* = 1.963, SE = 0.758, *p* = .010**

Interaction effects

1	momentary stress ~ ***helping * previous stress*** + freetime + ematime + (**helping** + freetime + ematime | subject)	*β* = -0.055, SE = 0.028, *p* =.055
2	momentary stress ~ ***helping * previous stress*** + freetime + ematime + (**helping** + freetime + ematime | subject)	*β* = -0.067, SE = 0.048, *p* =.164
1	momentary mood valence ~ ***helping * previous mood valence*** + freetime + ematime + (**helping** + freetime | subject)	*β* = -0.046, SE = 0.030, *p* =.128

2	momentary mood valence ~ ***helping * previous mood valence*** + freetime + ematime + (ematime + freetime | subject)	***β* = -0.117, SE = 0.050, *p* = .019**

Helping effort

Antecedents of helping effort

Burst	*Statistical model (R code)*	*Model output for the key predictor*
1	helping effort ~ **previous stress** + freetime + ematime + (**previous stress** + freetime | subject)	***β* = 0.067, SE = 0.028, *p* = .021**
2	helping effort ~ **previous stress** + freetime + ematime + (**previous stress** + freetime | subject)	*β* = 0.038, SE = 0.041, *p* = .352
1	helping effort ~ **previous mood valence** + freetime + ematime + (**previous mood valence** + freetime | subject)	*β* = -0.029, SE = 0.037, *p* = .434
2	helping effort ~ **previous mood valence** + freetime + ematime + (**previous mood valence** + freetime | subject)	*β* = -0.064, SE = 0.054, *p* = .237

Consequences of helping effort

1	momentary stress ~ **helping effort** + previous stress + freetime + ematime + (**helping effort** + freetime | subject)	***β* = 0.088, SE = 0.031, *p* = .005**
2	momentary stress ~ **helping effort** + previous stress + freetime + ematime + (previous stress | subject)	*β* = 0.026, SE = 0.049, *p* = .586
1	momentary mood valence ~ **helping effort** + previous mood valence + freetime + ematime + (**helping effort** + previous mood valence | subject)	*β* = -0.041, SE = 0.026, *p* = .124
2	momentary mood valence ~ **helping effort** + previous mood valence + freetime + ematime + (previous mood valence | subject)	*β* = -0.045, SE = 0.038, *p* = .235

Supplementary 5. Results for calmness and energetic arousal

Higher energetic arousal before and after helping

Antecedents of helping (preregistered). Higher energetic arousal in the previous data entry was associated with a greater likelihood of helping in the subsequent data entry in both study bursts (burst 1: $\beta = 0.011$, SE = 0.003, $p < .001$; burst 2: $\beta = 0.015$, SE = 0.006, $p = .016$). Calmness was not a significant predictor (*p*s > .495).

Consequences of helping (preregistered). Having helped since the previous data entry was associated with a higher energetic arousal in both study bursts (burst 1: $\beta = 2.405$, SE = 0.552, $p < .001$; burst 2: $\beta = 1.897$, SE = 0.871, $p = .030$). The analysis revealed no significant effect of helping on subsequent momentary calmness (*p*s > .380).

Interaction effects. We found a significant interaction between calmness from the previous data entry and helping on subsequent momentary calmness in burst 2 ($\beta = -0.121$, SE = 0.050, $p = .016$; burst 1: $p = .189$). However, the subsequent simple slope analysis was not significant. The interactions between perceived energetic arousal from the previous data entry and helping on subsequent energetic arousal were also not significant (*p*s > .068).

Table S3. Summary of significant relationships between helping, calmness, and energetic arousal.

Helping

Antecedents of helping		
1	**helping ~ previous energetic arousal** + freetime + ematime + (**previous energetic arousal** + freetime + ematime | subject)	**β = 0.011, SE = 0.003, p < .001**
2	**helping ~ previous energetic arousal** + freetime + ematime + (**previous energetic arousal** + freetime | subject)	**β = 0.015, SE = 0.006, p = .016**

Consequences of helping		
1	momentary energetic arousal ~ **helping** + previous energetic arousal + freetime + ematime + (**helping** + freetime | subject)	**β = 2.405, SE = 0.552, p < .001**
2	momentary energetic arousal ~ **helping** + previous energetic arousal + freetime + ematime + (**helping** + freetime | subject)	**β = 1.897, SE = 0.871, p = .030**
Interaction effects		
2	momentary calmness ~ ***helping * previous calmness*** + freetime + ematime + (**helping** + freetime | subject)	***β* = -0.121, SE = 0.050, *p* =.016**

More effortful helping associated with lower calmness and energetic arousal

Antecedents of helping effort (preregistered).

In study burst 2, lower perceived calmness from the previous data entry was associated with greater effort involved in subsequent helping (*β* = -0.112, SE = 0.049, *p* = .024; burst 1: *p* = .204). Energetic arousal was a not significant predictor (*p*s > .463).

Consequences of helping effort (not preregistered). We found that the greater the effort involved in helping was, the lower was the perceived calmness after helping in burst 1 (*β* = -0.063, SE = 0.029, *p* = .031; burst 2: p = .252) and the lower the energetic arousal in burst 2 (*β* = -0.082, SE = 0.039, *p* = .039; burst 1: *p* = .646).

Table S4. Summary of significant relationships between helping effort, calmness, and energetic arousal.

Helping effort

Antecedents of helping effort		
Burst	*Statistical model (R code)*	*Model output for the key predictor*

2	helping effort ~ **previous calmness** + freetime + ematime + (freetime | subject)	**β = -0.112, SE = 0.049, p = .024**
Consequences of helping effort		
1	momentary calmness ~ **helping effort** + previous calmness + freetime + ematime + (**helping effort** + previous calmness | subject)	**β = -0.063, SE = 0.029, p = .031**
2	momentary energetic arousal ~ **helping effort** + previous energetic arousal + freetime + ematime + (**helping effort** + previous energetic arousal | subject)	**β = -0.082, SE = 0.039, p = .039**

References

1. Caprara GV, Steca P, Zelli A, Capanna C. A New Scale for Measuring Adults' Prosocialness. Eur J Psychol Assess. 2005;21: 77–89. doi:10.1027/1015-5759.21.2.77
2. Klein EM, Brähler E, Dreier M, Reinecke L, Müller KW, Schmutzer G, et al. The German version of the Perceived Stress Scale – psychometric characteristics in a representative German community sample. BMC Psychiatry. 2016;16: 159. doi:10.1186/s12888-016-0875-9
3. Russell DW. UCLA Loneliness Scale (Version 3): Reliability, Validity, and Factor Structure. J Pers Assess. 1996;66: 20–40. doi:10.1207/s15327752jpa6601_2
4. Kroenke K, Spitzer RL, Williams JBW. The PHQ-9: Validity of a brief depression severity measure. J Gen Intern Med. 2001;16: 606–613. doi:10.1046/j.1525-1497.2001.016009606.x
5. Löwe BP. Gesundheitsfragebogen für Patienten PHQ-D: Manual; Komplettversion und Kurzform. Pfizer GmbH; 2001.
6. Kocalevent R-D, Hinz A, Brähler E. Standardization of the depression screener Patient Health Questionnaire (PHQ-9) in the general population. Gen Hosp Psychiatry. 2013;35: 551–555. doi:10.1016/j.genhosppsych.2013.04.006
